# A novel long-balloon dilator for endoscopic ultrasound-guided hepaticogastrostomy and bridging stenting with partial stent-in-stent method

**DOI:** 10.1055/a-2340-8631

**Published:** 2024-06-25

**Authors:** Fumitaka Niiya, Naoki Tamai, Jun Noda, Tetsushi Azami, Yuichi Takano, Fumiya Nishimoto, Masatsugu Nagahama

**Affiliations:** 1 26858 Division of Gastroenterology, Department of Internal Medicine, Showa University Fujigaoka Hospital, Yokohama, Japan

Endoscopic ultrasound-guided hepaticogastrostomy (EUS-HGS) is useful for the drainage of malignant hilar biliary obstructions 1 2 . The partial stent-in-stent (pSIS) method using the HGS route is helpful for draining the separated right intrahepatic bile duct; however, it is challenging because of long, tight bile duct strictures and difficulty in passing delivery systems through the stent mesh 3 . A novel balloon dilator ( Fig. 1 ) with a tapered tip and long dilation portion has recently become available in Japan. This report describes successful implementation of the pSIS method for hilar stenting of the right anterior bile duct (RAD) and right posterior bile duct (RPD) via the HGS route using such a novel long-type balloon dilator ( Video 1 ).

**Fig. 1:**
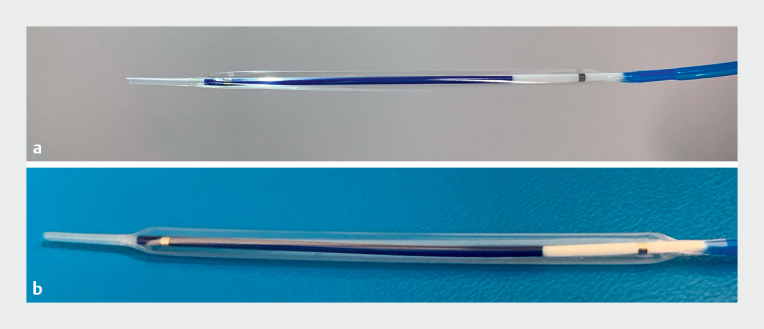
Features of the novel long-balloon dilator: **a** a tapered tip and **b** longer balloon length than in previously available devices.

Successful achievement of bridging stenting using the partial stent-in-stent method via the hepaticogastrostomy route, using a novel type of long-balloon dilator.Video 1

A 61-year-old man with unresectable gallbladder cancer, who had previously undergone duodenal stenting and EUS-HGS, was admitted with cholangitis. Computed tomography revealed dilatation of the RAD and RPD and multiple abscesses in the right hepatic lobe. Therefore, pSIS of the RAD and RPD via the HGS route was employed. Hydrophilic guidewires (0.025-inch) were advanced into the RAD and RPD in parallel using a double-lumen cannula (Uneven Double-Lumen Cannula; Piolax) ( Fig. 2 **a** ). A novel balloon dilator of diameter 3 mm and length 6 mm (REN IT; Kaneka) was inserted into the RPD, and the long RPD stricture was dilated in one session using the novel balloon dilator ( Fig. 2 **b** ). An uncovered self-expandable metal stent (SEMS) (Bilerush Selective; Piolax) was placed from the RPD to the left hepatic duct ( Fig. 2 **c** ). After insertion of a guidewire into the RAD through the stent mesh, the novel balloon dilator was advanced over the guidewire and the stent mesh was dilated. A second uncovered SEMS (Bilerush Selective) was deployed from the RAD to the left hepatic duct ( Fig. 2 **d** ). This unique long-balloon dilator allowed effective implementation of the pSIS method via the HGS route.

**Fig. 2:**
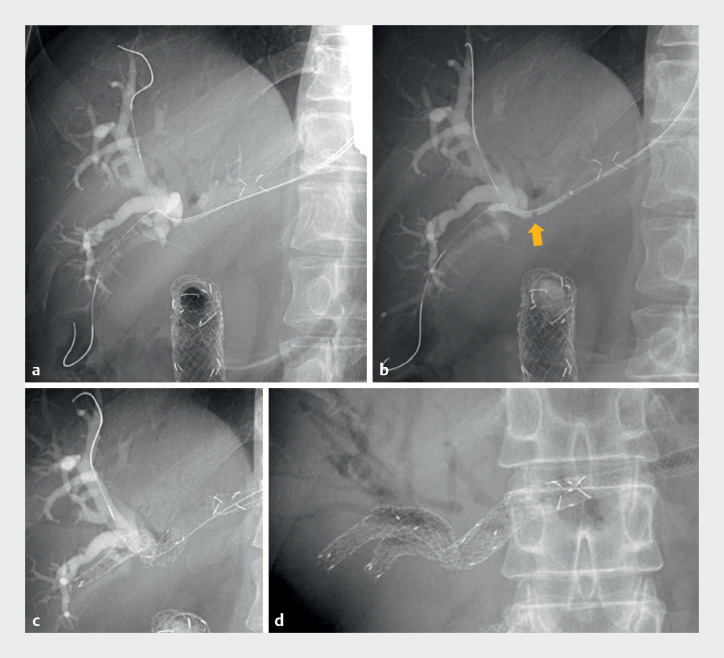
**a** A 0.025-inch guidewire was placed in B8 and another in B6, in parallel. **b** The B6 stricture was dilated in one session using the novel long-balloon dilator (arrow). **c** An uncovered self-expandable metal stent was placed between the posterior bile duct and the left hepatic duct. **d** A second uncovered self-expandable metal stent was deployed through the first metal stent mesh between B5 and the left hepatic duct.

Endoscopy_UCTN_Code_TTT_1AS_2AD

## Notes

Endoscopy E-Videos https://eref.thieme.de/e-videos
*E-Videos* is an open access online section of the journal *Endoscopy* , reporting on interesting cases and new techniques in gastroenterological endoscopy. All papers include a high-quality video and are published with a Creative Commons CC-BY license. Endoscopy E-Videos qualify for HINARI discounts and waivers and eligibility is automatically checked during the submission process. We grant 100% waivers to articles whose corresponding authors are based in Group A countries and 50% waivers to those who are based in Group B countries as classified by Research4Life (see: https://www.research4life.org/access/eligibility/ ). This section has its own submission website at https://mc.manuscriptcentral.com/e-videos .
